# Ecological association between Grindr user characteristics and HIV prevalence in Malaysia: a cross-sectional study

**DOI:** 10.1186/s13104-026-07920-5

**Published:** 2026-06-19

**Authors:** Hakimi Kassim, Kamarul Ismail, Md Enamul Huq, Hein Htet Aung, Ethan Trey Cardwell, Fabian Yuh Shiong Kong

**Affiliations:** 1 Department of Bioengineering, Imperial College London, London, SW7 2AZ UK; 2 Department of Biology, Faculty of Science and Mathematics, Sultan Idris University of Education, Perak, 35900 Malaysia; 3 Department of Geography and Environment, Faculty of Human Sciences, Sultan Idris University of Education, Perak, 35900 Malaysia; 4 Melbourne School of Population and Global Health, The University of Melbourne, Victoria, 3053 Australia

**Keywords:** HIV epidemiology, Men who have sex with men, Geosocial networking application, Spatial analysis, Sexual network

## Abstract

**Objective:**

Men who have sex with men in Malaysia face a high HIV burden, but demographic, behavioral, and digital health data remain limited by social constraints. This nationwide, cross-sectional ecological study utilized a grid-based geographic sampling method to systematically collect publicly accessible Grindr profiles across all Malaysian states and federal territories. Population-normalized Grind user rates, profile characteristics, and regional people living with HIV rates were analyzed using Spearman’s rank-order correlation to evaluate whether geosocial networking platform data offer insights into regional HIV epidemiology.

**Results:**

A significant positive correlation was observed between population-normalized Grindr user rates and PLHIV rates (*r*_*s*_ = 0.5821, *P* = 0.0252). While unadjusted analyses showed nominal positive correlations between regional PLHIV rates and specific profile characteristics, none maintained significance following false discovery rate correction (*Q* = 5%). This lack of post-correction significance is driven by the limited statistical power of the regional sample size (*n* = 16) and high percentages of profile non-disclosure. Thus, while grid-based sampling systematically assesses digital user density, individual profile characteristics cannot reliably indicate regional HIV prevalence when data omissions are widespread.

**Supplementary Information:**

The online version contains supplementary material available at https://doi.org/10.1186/s13104-026-07920-5.

## Introduction

According to the 2025 Malaysian Global AIDS Monitoring (GAM) report, men who have sex with men (MSM) are disproportionately impacted by HIV. This signifies a transition from injection drug use and sex work to same-sex sexual transmission [1]. The majority of new HIV cases are among MSM, with significant geographic variation across states and federal territories (FDs). Despite this transition, empirical data regarding MSM behavior remains scarce. HIV diagnoses are recorded through sexual health clinic screening programs, but little is known about the impact of digital platforms on HIV epidemiology, particularly in settings with social and legal constraints. Understanding geosocial networking application users and their ecological association with PLHIV rates may help explain regional differences without implying individual risk.

Geosocial same-sex networking applications such as Grindr offer real-time and location-based interaction for partner-seeking based on proximity and profile information [2]. Grindr has been widely used in sexual health research that provides insights into behaviors and risks associated with HIV and other sexually transmitted infections (STIs). Previous studies have recruited participants through in-app advertisements leading to external surveys [2] or through cross-sectional surveys employing community outreach and online platforms [3]. Moreover, another independent study investigated self-reported profile characteristics, including preferences for sexual positions and the disclosure of HIV status [4].

In-app observational methodologies can generate population-level insights into MSM communities and their significance for HIV and STI prevention [5]. In addition to spatial epidemiology, Grindr serves as a recruitment platform for implementation and formative studies involving MSM, including HIV prevention in high-stigma environments like Malaysia [6]. This platform enables population-level analysis and necessitates integrating spatial distribution with behavioral traits. In our study, user profiles on Grindr were analyzed across all Malaysian states and FDs utilizing a grid-based sampling approach. This study evaluates the correlation of Grindr user characteristics with PLHIV rates at the population level.

## Methods

**Fig. 1:**
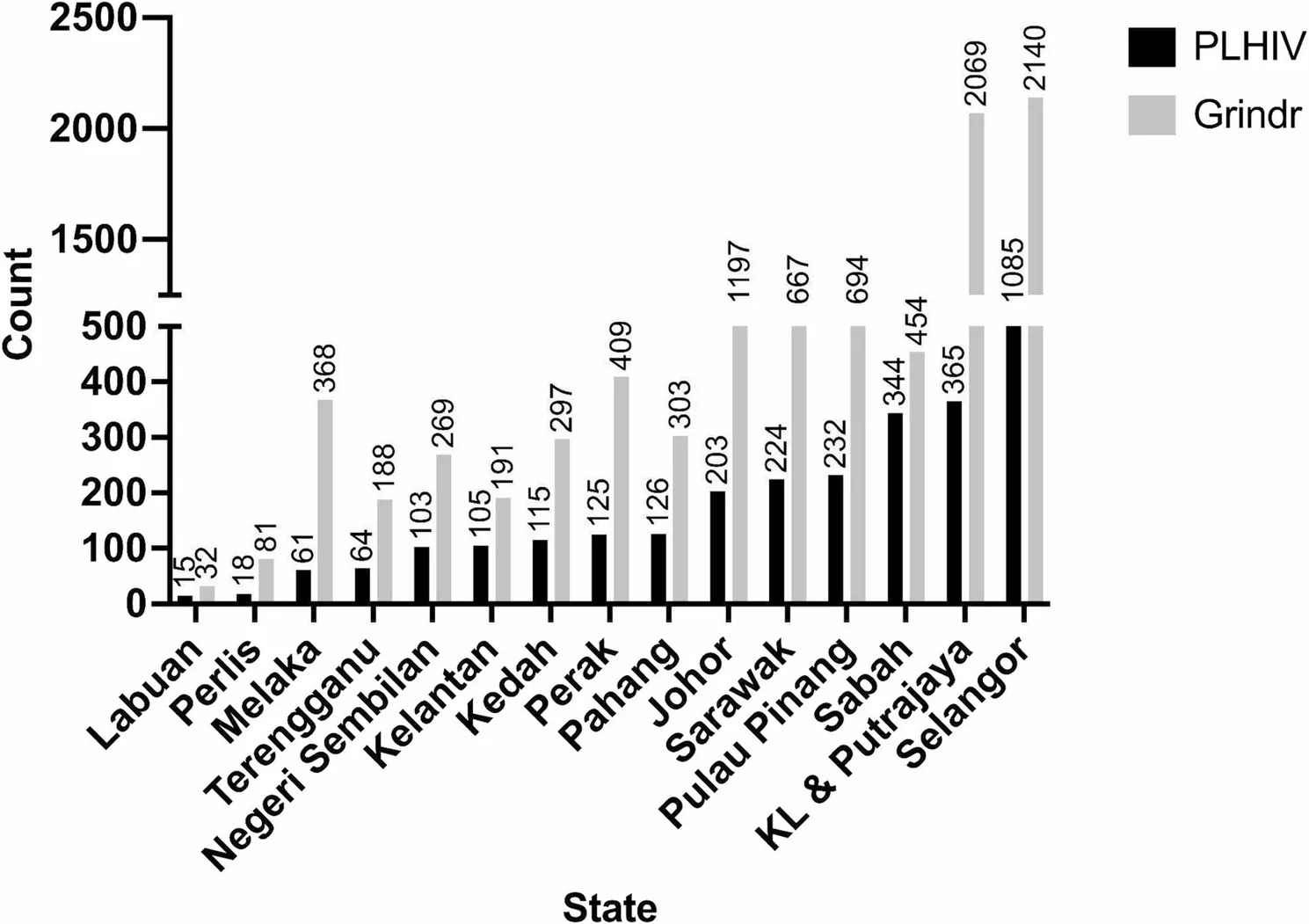
Total number of PLHIV reported in the Malaysia Global AIDS Monitoring Report and distribution of online Grindr users captured during a cross-sectional sampling period (Saturday, 14 December 2024, 12:00–18:00) using a grid-based sequential centroid approach. A statistically significant positive correlation was observed between Grindr user rates and PLHIV rates at the population level (Spearman’s *r*_*s*_ = 0.5821, *P* = 0.0252)

This nationwide cross-sectional study elucidated the correlation between Grindr user characteristics and PLHIV rates at the population level. The study size was determined by the inclusion of all 13 Malaysian states and 3 FDs as the unit of ecological analysis. Based on the 2025 GAM report, the number of PLHIV varies across Malaysian states and FDs, shown in Fig. 1 [1]. All states and FDs were categorized into small (Perlis, Penang, Melaka and Labuan), medium (Negeri Sembilan, Selangor, Kedah, Terengganu, Kelantan, Johor, Perak, and Pahang) and large (Sabah and Sarawak) categories based on land area to ensure sufficient geographical coverage and resolutions. The grid sizes and their corresponding centroid radii were categorized as follows: (1) small states: 4 km grid (2 km radius); (2) medium states: 8 km grid (4 km radius); and (3) large states: 12 km grid (6 km radius). The coordinates of centroids derived from grids mapped onto Google Earth application served as data collection points.

For data collection, a Grindr account was signed up exclusively for this study and deleted following the completion of data collection. This study adopted the methodologies described in the previous studies [5, 7, 8]. Only publicly accessible information from user profiles was recorded, eliminating the need for individual informed consent. Data was collected remotely on Saturday, December 14, 2024, between 12:00 p.m. and 6:00 p.m. via a mobile device, a timeframe selected to capture peak usage based on pilot testing. Geographic sampling involved entering predefined GIS coordinates into Grindr to capture users within each centroid radius. To ensure geographic accuracy, only “online” profiles were included, while “recently active” profiles were excluded to ensure users were currently located within the centroid radius. Publicly available profile details were recorded as presented without verification. Variables were categorised as single- or multi-select. Single-select variables included age, height, weight, body type, ethnicity, gender, sexual position identity, relationship status, self-reported HIV status, and HIV testing history. Multi-select variables included sexual subculture identity, app use intentions, preferred meeting place, pronouns, NSFW preferences, health practices, vaccination status, and linked social media. To ensure methodological reliability and consistency, data collection was conducted using predefined GIS coordinates, standardized centroid radii based on state size, and a fixed sampling timeframe. No personally identifiable information was recorded, and data was stored in a password-protected Microsoft Excel file.

Grindr user counts, user variables, and PLHIV counts were normalised to state population size and analysed as population-level rates. Normality was assessed using the Shapiro-Wilk test (Supplementary Tables 1 and 2). Spearman’s rank-order correlation was first employed to evaluate the strength and direction of associations among Grindr users, user variables, and PLHIV rates, generating correlation coefficients (*r*_*s*_) and unadjusted *P*-values (Supplementary Table 3). The threshold for initial statistical significance was set at *P* < 0.05. To account for multiple comparisons and control the inflating risk of Type I errors across multiple user variables, a False Discovery Rate (FDR) analysis was subsequently performed using the Benjamini and Hochberg method with a desired FDR (*Q*) of 5%. Associations meeting this FDR threshold, represented by adjusted *Q*-values (*Q* < 0.05), were considered statistically significant (Supplementary Table 3). All analyses were conducted using GraphPad Prism (version 9.5.1).

## Results

### Positive correlation between Grindr user rates and PLHIV rates across Malaysian states and federal territories

According to the Malaysia AIDS Monitoring Report 2025, there were 3,185 PLHIV and 9,359 Grindr users across all states and FDs (Fig. 1). Counts varied substantially by region, with higher numbers in Selangor, Kuala Lumpur and Putrajaya, Sabah, Johor, and Sarawak, and lower counts in Perlis, Terengganu, and Labuan. A statistically significant positive association was observed between population-normalised Grindr user rates and PLHIV rates at the population level (Spearman’s *r*_*s*_ = 0.5821, *P* = 0.0252).

### Characteristics of Grindr users across Malaysian states and federal territories

The distribution of demographic, behavioural, digital interaction, and health-related features across Malaysian states and FDs, alongside their Spearman rank-order correlations (*r*_*s*_), unadjusted *P*-values, and FDR-adjusted *Q*-values with PLHIV rates at the population level, are detailed in Supplementary Table 3. Notably, non-disclosure rates were high across nearly all user variables. Demographic and physical variables were largely undisclosed, including age (72.3%), height (73.6%), weight (77.5%), body type (74.3%), ethnicity (69.0%), and gender (82.9%). High non-disclosure rates also persisted for crucial behavioral and clinical indicators, such as relationship status (84.7%), sexual position identity (59.4%), and HIV status (74.6%). Prevention engagement practices, including condom use, HIV pre-exposure prophylaxis (PrEP), doxycycline post-exposure prophylaxis (doxyPEP), and self reported undetectable status, were entirely not reported across all profiles, resulting in uniform responses that precluded meaningful variation for statistical analysis.

In the unadjusted analysis, nominal positive correlations (*P* < 0.05) were initially observed between several user characteristics and PLHIV rates at the population level. These included specific age brackets (21 to 30 and 31 to 40 years), body types (toned and muscular), the man gender category, and Asian ethnicity. Similar unadjusted correlations emerged for open relationship statuses, top and bottom sexual positions, certain subcultures (daddy, twink, and discreet), and intentions centered on hookups or relationships. Stronger correlations were observed for private meeting venue preferences including “my place” and “your place”, alongside the non-disclosure of HIV status and testing history. Crucially, however, after applying the Benjamini and Hochberg method to control the FDR at *Q* = 5% across these multiple comparisons, no evaluated variables maintained statistical significance. Following adjustment, all corresponding *Q*-values exceeded the significance threshold (*Q* ≥ 0.1099) (Supplementary Table 3). Ultimately, no independent demographic, behavioral, or digital interaction characteristic of Grindr users demonstrated a robust statistical correlation with regional PLHIV rates once the inflation of Type I errors was strictly controlled.

## Discussion

An ecological correlation was initially observed between higher regional HIV burdens and greater numbers of Grindr users across Malaysian states and FDs. Similar population-level patterns between geosocial application density and HIV or sexually transmitted infection distributions have been reported in North America and Europe [7, 8]. As HIV diagnosis and reporting are heavily influenced by urbanization, mobility, and the geographic concentration of MSM communities reflected in application density, these findings do not imply individual transmission risk. To capture these geographic distributions, this study utilized a novel grid-based sampling approach, which offers a systematic method for quantifying app user density across large geographic areas.

Prior to multiple testing correction, several demographic, behavioral, and digital characteristics, such as specific age groups, body types, open relationships, sexual positions, subcultures, private venue preferences, and the non disclosure of HIV metrics, demonstrated nominal positive correlations with regional PLHIV rates. Notably, literature corroborates these unadjusted variables as established drivers of MSM HIV vulnerability, including young age [9], idealized body types [10–13], non-monogamous relationship [14, 15], receptive sexual roles [16, 17], sexual subculture identities including ‘twink’, ‘discreet’ and ‘daddy’ [18, 19], and hookup or relationship-seeking intentions [20, 21] or private encounter venues [22]. Furthermore, identity concealment driven by stigma frequently underpins the widespread non-disclosure of health metrics within socially constrained settings [23]. Crucially, however, in the adjusted model, none of these variables maintained statistical significance. A key structural finding explaining this outcome is the highly constrained regional sample size (*n* = 16) working in tandem with a substantially high percentage of non-disclosure observed across nearly all evaluated profile variables. This extensive omission of user attributes markedly reduced statistical power and precluded meaningful variation for regional correlation analysis.

The widespread non-disclosure of health practices, testing histories, and health metrics may reflect socio-cultural constraints, systemic stigma, and concealment requirements in this setting. Consequently, to avoid an ecological fallacy, the unadjusted trends observed in this population must not be interpreted as indicators of individual behavioral risk, partner selection preferences, or sexual network vulnerabilities. Instead, they likely reflect demographic subgroup sizes, network connectivity, and regional differences in information disclosure rather than direct transmission probabilities. These post-correction results demonstrate that individual profile characteristics on geosocial applications cannot serve as robust indicators of regional HIV prevalence when data omissions are widespread. Future research utilizing grid sampling should incorporate strategies to account for high data non-disclosure when analyzing ecological health outcomes through digital health metrics.

## Conclusion

This study demonstrates that an innovative, grid-based sampling methodology provides a highly systematic and novel approach for capturing geosocial networking application data and user density across large geographic areas. While multiple testing corrections revealed that individual profile characteristics did not maintain robust statistical correlations with regional PLHIV rates due to high percentages of non-disclosure, these aggregate metrics still offer a valuable framework for examining digital population density. Although causality of HIV transmission cannot be inferred, these findings highlight the necessity for geographically derived surveillance data. To enhance robustness and address widespread data omission, future research could employ longitudinal and multi platform methodologies to inform more adaptive, context specific public health strategies in scenarios where data collection and behavioral surveillance are constrained.

### Limitations

Despite the novelty of using a grid-based sampling method to systematically capture geosocial application data across all Malaysian administrative regions, several limitations remain. Cross-sectional ecological design of this study precludes the establishment of causal links between Grindr user characteristics and regional HIV rate, therefore population-level findings cannot be used to estimate individual risk. The six-hour data collecting window on a given day also reduces temporal representativeness. Since all variables were self-reported and unverified, the lack of HIV-positive reports suggests a reporting bias. Additionally, many profile variables showed high rates of non-disclosure, which may limit representativeness and introduce self-selection bias toward users who were more willing to disclose personal information. Importantly, all correlation analyses were conducted at the regional level using only 13 states and 3 FDs (*n* = 16), resulting in limited statistical power and potentially unstable correlation estimates that may be disproportionately influenced by a small number of regions.

## Supplementary Information


Supplementary Material 1.


## Data Availability

The datasets produced and examined in this study can be obtained from the corresponding author upon a reasonable request.

## Abbreviations

doxyPEP: Doxycycline post-exposure prophylaxis
FD: Federal territory
FDR: False discovery rate
MSM: Men who have sex with men
PLHIV: People living with HIV
PrEP: HIV pre-exposure prophylaxis
STI: Sexually transmitted infection
