# Supplementary material for: Ecological association between Grindr user characteristics and HIV prevalence in Malaysia: a cross-sectional study
Source: BMC Res Notes. 2026 Jun 19;19:337. doi: 10.1186/s13104-026-07920-5 (PMC13523420; doi:10.1186/s13104-026-07920-5)
Supplement: Supplementary file 1 [file 13104_2026_7920_MOESM1_ESM.pdf]

Supplementary Tables

Supplementary Table 1. Shapiro-Wilk normality test for Grindr user rates and PLHIV rates across Malaysian states and federal territories (n = 16).

**Variable**	**W**	***P* value**	**Passed normality test (alpha = 0.05)?**	***P* value summary**
Grindr	0.6940	0.0002	No	***
PLHIV	0.8552	0.0206	No	*

Note: * $P < 0.05$; *** $P < 0.001$

Supplementary Table 2. Shapiro-Wilk normality test for Grindr user variable rates across Malaysian states and federal territories (n = 16).

Variable	Category	W	*P*-value	Passed normality test (alpha = 0.05)?	*P*-value summary
Age	Age (18-20)	0.9114	0.1421	Yes	not significant
	Age (21-30)	0.6895	0.0002	No	***
	Age (31-40)	0.6796	0.0002	No	***
	Age (41-50)	0.8668	0.0302	No	*
	Non-disclosure	0.7447	0.0008	No	***
Height	Height (161-170)	0.6541	<0.0001	No	****
	Height (171-180)	0.7841	0.0024	No	**
	Height (non-disclosure)	0.7140	0.0004	No	***
	Weight (51-60)	0.7191	0.0004	No	***
	Weight (61-70)	0.7438	0.0008	No	***
	Weight (71-80)	0.8781	0.0445	No	*
	Non-disclosure	0.6676	0.0001	No	***
Body type	Slim	0.9082	0.1272	Yes	not significant
	Stocky	0.9281	0.2553	Yes	not significant
	Average	0.6661	0.0001	No	***
	Toned	0.6651	0.0001	No	***
	Muscular	0.7465	0.0008	No	***
	Large	0.7539	0.0010	No	***
	Non-disclosure	0.7116	0.0003	No	***
Ethnicity	Asian	0.6746	0.0001	No	***
	Mixed	0.8207	0.0069	No	**
	White	0.6786	0.0001	No	***
	Non-disclosure	0.7072	0.0003	No	***
Gender	Man	0.6478	<0.0001	No	****
	Cis-man	0.7928	0.0030	No	**
	Non-binary	0.7291	0.0005	No	***
	Trans women	0.6841	0.0002	No	***
	Non-disclosure	0.6920	0.0002	No	***
Relationship status	Single	0.8500	0.0174	No	*

	Open relationship	0.6835	0.0002	No	***
	Married	0.7149	0.0004	No	***
	Partnered	0.8406	0.0128	No	*
	Non-disclosure	0.6932	0.0002	No	***
Sexual position identity	Top	0.9305	0.278	Yes	not significant
	Bottom	0.7008	0.0003	No	***
	Versatile	0.7352	0.0006	No	***
	Versatile Top	0.8861	0.0586	Yes	not significant
	Versatile Bottom	0.8452	0.0148	No	*
	Side	0.7777	0.0019	No	**
	Non-disclosure	0.7145	0.0004	No	***
Sexual subculture identity	Discreet	0.6912	0.0002	No	***
	Clean-cut	0.8928	0.074	Yes	not significant
	Twink	0.6893	0.0002	No	***
	Daddy	0.6735	0.0001	No	***
	Jock	0.7734	0.0017	No	**
	Bear	0.7410	0.0007	No	***
	Trans	0.5668	<0.0001	No	****
	Non-disclosure	0.7455	0.0008	No	***
Application use intention	Hookups	0.7760	0.0018	No	**
	Relationship	0.6980	0.0002	No	***
	Friends	0.7705	0.0016	No	**
	Dates	0.7155	0.0004	No	***
	Chat	0.7034	0.0003	No	***
	Networking	0.6840	0.0002	No	***
	Non-disclosure	0.8644	0.0279	No	*
Meeting venue	My place	0.8268	0.0083	No	**
	Your place	0.7919	0.0029	No	**
	Bar	0.7499	0.0009	No	***
	Coffee shop	0.6792	0.0002	No	***
	Restaurant	0.7394	0.0007	No	***
	Non-disclosure	0.6662	0.0001	No	***
Pronoun	He/him/his	0.7879	0.0034	No	**

	She/her/hers	0.4840	<0.0001	No	****
	Ask me	0.7396	0.0007	No	***
	Use my name only	0.7671	0.0014	No	**
	Non-disclosure	0.7064	0.0003	No	***
Accept NSFW picture	Never	0.7071	0.0003	No	***
	Not at first	0.8015	0.0038	No	**
	Yes, please	0.9609	0.7083	Yes	not significant
	Non-disclosure	0.7223	0.0004	No	***
Social links	Facebook	0.8642	0.0278	No	*
	Instagram	0.7292	0.0005	No	***
	X	0.7778	0.0019	No	**
	Non-disclosure	0.7030	0.0003	No	***
HIV status	Negative	0.9329	0.3013	Yes	not significant
	Negative, on PrEP	0.8559	0.0211	No	*
	Non-disclosure	0.6546	<0.0001	No	****
HIV testing history	Last 6 months	0.5817	<0.0001	No	****
	Beyond 6 months	0.7198	0.0004	No	***
	Non-disclosure	0.7481	0.0009	No	***
Health practices	Non-disclosure	0.6940	0.0002	No	***
Vaccinations	Covid-19	0.9633	0.7495	Yes	not significant
	Meningitis	0.8677	0.0313	No	*
	Monkeypox	0.5767	<0.0001	No	****
	Non-disclosure	0.6017	<0.0001	No	****

Note: * $P < 0.05$; ** $P < 0.01$; *** $P < 0.001$; **** $P < 0.0001$

Supplementary Table 3. Spearman correlation and FDR correction of Grindr user variable rates involving 9359 Grindr users across Malaysian states and FDs (n = 16).

Demographic and structural composition						
Variable	Category	Count (n)	Count (%)	Spearman (r_s)	*P-value*	*Q-value*
Age	18-20<br>21-30<br>31-40<br>41-50<br>Non-disclosure	159<br>1556<br>765<br>117<br>6762	1.7<br>16.6<br>8.2<br>1.3<br>72.3	0.3181<br>0.5321<br>0.5464<br>0.4290<br>N/A	0.2460<br>0.0438<br>0.0377<br>0.1114<br>N/A	0.2884<br>0.1265<br>0.1225<br>0.1931<br>N/A
Height (cm)	161-170<br>171-180<br>Non-disclosure	1179<br>1294<br>6886	12.6<br>13.8<br>73.6	N/A<br>N/A<br>N/A	N/A<br>N/A<br>N/A	N/A<br>N/A<br>N/A
Weight (kg)	51-60<br>61-70<br>71-80<br>Non-disclosure	360<br>934<br>816<br>7249	3.8<br>10<br>8.7<br>77.5	N/A<br>N/A<br>N/A<br>N/A	N/A<br>N/A<br>N/A<br>N/A	N/A<br>N/A<br>N/A<br>N/A
Body type	Slim<br>Stocky<br>Average<br>Toned<br>Muscular<br>Large<br>Non-disclosure	603<br>168<br>288<br>755<br>381<br>210<br>6954	6.4<br>1.8<br>3.1<br>8.1<br>4.1<br>2.2<br>74.3	0.2821<br>0.4301<br>0.2038<br>0.5536<br>0.6321<br>0.3857<br>N/A	0.3073<br>0.1112<br>0.4631<br>0.0349<br>0.0134<br>0.1566<br>N/A	0.3400<br>0.1931<br>0.4722<br>0.1210<br>0.1099<br>0.2199<br>N/A
Ethnicity	Asian<br>Mixed<br>White<br>Non-disclosure	2619<br>184<br>98<br>6458	28<br>2<br>1<br>69	0.6250<br>0.4126<br>0.2576<br>N/A	0.0148<br>0.1268<br>0.3502<br>N/A	0.1099<br>0.1966<br>0.3716<br>N/A
Gender†	Man	1317	14.1	0.5250	0.0471	0.1289

	Cis-man<br>Non-binary<br>Trans woman<br>Non-disclosure	205<br>38<br>43<br>7756	2.2<br>0.4<br>0.5<br>82.9	0.4433<br>0.4300<br>0.4649<br>N/A	0.0991<br>0.1105<br>0.0825<br>N/A	0.1909<br>0.1931<br>0.1722<br>N/A
**Sexual network configuration**						
Variable	Category	Count (n)	Count (%)	Spearman (r_s)	*P-value*	*Q-value*
Relationship status	Single<br>Open<br>relationship<br>Married<br>Partnered<br>Dating<br>Engaged<br>Exclusive<br>Non-disclosure	899<br>432<br>36<br>61<br>0<br>0<br>0<br>7931	9.6<br>4.6<br>0.4<br>0.7<br>0<br>0<br>0<br>84.7	0.5000<br>0.5893<br>0.4110<br>0.3164<br>0<br>0<br>0<br>N/A	0.0602<br>0.0232<br>0.1293<br>0.2496<br>0<br>0<br>0<br>N/A	0.1361<br>0.1140<br>0.1966<br>0.2884<br>0<br>0<br>0<br>N/A
Sexual position identity	Top<br>Bottom<br>Versatile<br>Versatile top<br>Versatile<br>bottom<br>Side<br>Non-disclosure	1074<br>1418<br>882<br>173<br>142<br>110<br>5560	11.5<br>15.2<br>9.4<br>1.8<br>1.5<br>1.2<br>59.4	0.5679<br>0.6500<br>0.3680<br>0.4072<br>0.2990<br>0.3892<br>N/A	0.0297<br>0.0105<br>0.1779<br>0.1323<br>0.2763<br>0.1524<br>N/A	0.1188<br>0.1099<br>0.2372<br>0.1966<br>0.3123<br>0.2199<br>N/A
Sexual subculture identity†	Discreet<br>Clean-cut<br>Twink<br>Geek<br>Daddy<br>Rugged<br>Jock	1800<br>526<br>657<br>0<br>719<br>0<br>528	19.2<br>5.6<br>7<br>0<br>7.7<br>0<br>5.6	0.6000<br>0.2536<br>0.6714<br>0<br>0.5786<br>0<br>0.3382	0.0203<br>0.3607<br>0.0061<br>0<br>0.0263<br>0<br>0.2171	0.1140<br>0.3751<br>0.1099<br>0<br>0.1140<br>0<br>0.2688

	Bear	497	5.3	0.2752	0.3180	0.3445
	Leather	0	0	0	0	0
	Trans	32	0.3	0.3466	0.2052	0.2603
	Otter	0	0	0	0	0
	Poz	0	0	0	0	0
	Sober	0	0	0	0	0
	Non-disclosure	4600	49.2	N/A	N/A	N/A
Application use intention[†]	Hookups	1643	17.6	0.6143	0.0170	0.1105
	Relationship	974	10.3	0.5393	0.0406	0.1242
	Friends	724	7.7	0.5107	0.0543	0.1345
	Dates	777	8.2	0.5036	0.0582	0.1361
	Chat	857	9.1	0.3607	0.1870	0.2431
	Networking	871	9.2	0.3179	0.2480	0.2884
	Non-disclosure	3613	38.2	N/A	N/A	N/A
**Encounter and digital interaction context**						
Variable	Category	Count (n)	Count (%)	Spearman (r_s)	*P-value*	*Q-value*
Meeting venue[†]	My place	1254	13.4	0.7500	0.0019	0.0988
	Your place	1073	11.5	0.6607	0.0089	0.1099
	Bar	950	10.2	0.5164	0.0515	0.1339
	Coffee shop	1077	11.5	0.4643	0.0834	0.1722
	Restaurant	1747	18.7	0.1643	0.5580	0.5580
	Non-disclosure	3258	34.8	N/A	N/A	N/A
Pronoun[†]	He/him/his	1374	14.7	N/A	N/A	N/A
	She/her/hers	20	0.2	N/A	N/A	N/A
	Ask me	22	0.2	N/A	N/A	N/A
	Use my name only	118	1.3	N/A	N/A	N/A
	Non-disclosure	7825	83.6	N/A	N/A	N/A
Accept	Never	427	4.6	N/A	N/A	N/A

NSFW picture	Not at first<br>Yes, please<br>Non-disclosure	313<br>234<br>8385	3.3<br>2.5<br>89.6	N/A<br>N/A<br>N/A	N/A<br>N/A<br>N/A	N/A<br>N/A<br>N/A
Social links†	Facebook<br>Instagram<br>X<br>Spotify<br>Non-disclosure	366<br>555<br>512<br>0<br>7926	3.9<br>5.9<br>5.5<br>0<br>84.7	N/A<br>N/A<br>N/A<br>N/A<br>N/A	N/A<br>N/A<br>N/A<br>N/A<br>N/A	N/A<br>N/A<br>N/A<br>N/A<br>N/A
**HIV-related disclosure and prevention engagement**						
Variable	Category	Count (n)	Count (%)	Spearman (r_s)	*P-value*	*Q-value*
HIV status	Negative<br>Negative, on PrEP<br>Positive<br>Positive, Undetectable<br>Non-disclosure	1493<br>880<br>0<br>0<br><br>6986	16<br>9.4<br>0<br>0<br><br>74.6	0.4179<br>0.3821<br>0<br>0<br><br>0.6357	0.1227<br>0.1607<br>0<br>0<br><br>0.0128	0.1966<br>0.2199<br>0<br>0<br><br>0.1099
HIV testing history	Last 6 months<br>Beyond 6 months<br>Non-disclosure	502<br>1322<br>7535	5.4<br>14.1<br>80.5	0.4218<br>0.4607<br>0.5571	0.118<br>0.0861<br>0.0335	0.1966<br>0.1722<br>0.1210
Health practices†	Condoms<br>I'm on doxyPEP<br>I'm on PrEP<br>I'm HIV undetectable<br>Non-disclosure	0<br>0<br>0<br>0<br><br>9359	0<br>0<br>0<br>0<br><br>100	0<br>0<br>0<br>0<br><br>0.5821	0<br>0<br>0<br>0<br><br>0.0252	0<br>0<br>0<br>0<br><br>0.1140

Vaccinations†	Covid-19<br>Meningitis<br>Monkeypox<br>Non-disclosure	3040<br>176<br>36<br>6107	32.5<br>1.9<br>0.4<br>65.3	N/A<br>N/A<br>N/A<br>N/A	N/A<br>N/A<br>N/A<br>N/A	N/A<br>N/A<br>N/A<br>N/A

Note: Multi-select variables are denoted with superscript dagger (†). N/A indicates variables not considered key contributors to HIV transmission risk.
